# Arabic Validation of the Amsterdam Preoperative Anxiety and Information Scale

**DOI:** 10.7759/cureus.28004

**Published:** 2022-08-14

**Authors:** Sulaman ALMesned, Abdulmonem A Alsalhi, Said Abdelsalam, Lubna Alquwayfili, Thamer K Alharbi, Fahad Almazyad, Ali Alshaya, Saleh Aldubayyan, Abdullah s Alkhumayri, Roaa Almazyad

**Affiliations:** 1 Anesthesiology, Qassim University, Buraydah, SAU; 2 Anaesthesiology, Qassim University, Buraydah, SAU; 3 Anaesthesiology, King Fahad Specialist Hospital, Buraydah, SAU; 4 Surgery, King Fahad Specialist Hospital, Buraydah, SAU

**Keywords:** genral surgery, apais, anxiety scale, preoperative anxiety, `anesthesia

## Abstract

Background

*Preoperative anxiety* is a frequent and challenging problem that may impact a patient's postoperative pain and satisfaction. The level of a patient's anxiety needs to be assessed through a valid and reliable instrument to prevent and treat preoperative anxiety effectively. One such reliable measurement scale is Amsterdam Preoperative Anxiety and information scale, which is based on a self-reported questionnaire but is still not validated in Arabic.

Objective

To validate the Arabic Version of the Amsterdam Preoperative Anxiety and Information Scale (APAIS) for assessing preoperative anxiety in the Arabic population.

Methods

A cross-sectional study was conducted to translate and evaluate the validity of the APAIS in the Arabic version. The targeted population was Saudi adults undergoing surgery at the King Fahad Hospital in the Qassim region of Saudi Arabia. One hundred hospital patients were recruited and given the APAIS questionnaire to collect the data. The ethical considerations have been appropriately followed to protect the privacy of the patient's history. The collected data was qualitative and quantitative, which were analyzed using Statistical Package for the Social Sciences (SPSS).

Results

The questionnaire showed high internal consistency on the anxiety scale (Cronbach's alpha: 0.851) and a strong correlation between age, chronic diseases, and surgery. While Cronbach's alpha for the information scale is 0.827. The gold standard curve between the worried and afraid two variables showed good efficiency during the configuration. In addition, the Confirmatory Factor Analysis (CFA) model of the Arabic version is a two-factor model to evaluate the validity of the Arabic version.

Conclusion

The Arabic Version of the Amsterdam Preoperative Anxiety and information scale (APAIS) is a valid and reliable instrument for assessing preoperative anxiety. Using this validated scale for Arabic patients is feasible and shows promising results.

## Introduction

Preoperative anxiety is frequently perceived by patients undergoing surgical procedures. It is described as excessive worry, nervousness, and uneasiness before surgery [1]. When it is excessively felt, the anxiety could be exhibited behaviorally and psychologically, ranging from postoperative pain and hypertension to depression and arrhythmias [2]. Higher anxiety levels may influence preoperative anesthesia management, making it more difficult to omit the sympathetic system's overstimulation, thus influencing pre- medications and the induction of anesthesia [3,4].

Preoperative anxiety produces a physiological response that prepares the body for a perceived danger and is linked to a negative mental state. This could have a detrimental impact on the success of the surgical intervention. The stress response, which includes the release of catecholamines, sympathetic hyperactivity, hyper-metabolism, neuroendocrine changes, electrolyte abnormalities, and immunological modifications, is likely to occur when anxiety levels rise before an intervention. High preoperative anxiety patients need a higher dosage of analgesic and anesthetic medications. This often prolongs hospital stays, increases the chance of readmission after surgery, and enhances morbidity and mortality. These issues demonstrate the need to evaluate and treat preoperative anxiety, supporting the regular provision of preoperative anxiolytics to all surgical patients.

Many instruments measure a patient's level of preoperative anxiety [5,6]. The Amsterdam Preoperative Anxiety and Information Scale (APAIS) is a simple and reliable questionnaire that can be completed in a few minutes. Anesthetists use APAIS to improve patient care by assessing patients and the degree of information they require to reduce their anxiety. APAIS is translated into many languages, including English, Japanese, French, German, Italian, Spanish, and Chinese [4-10].

The Italian version of APAIS was validated in a 2017 study [8], first translated into APAIS Italian, then administered to the patients. It has two factors; anxiety and the need for information. As observed in the Dutch Version, the identical items loaded with the same factors as the original questionnaire. Internal consistency was explored by calculating Cronbach's alpha coefficient, the APAIS anxiety subscale, and the total APAIS scores showed high reliability; however, unlike the original version, the Italian APAIS need for information subscale had a high internal consistency. This result perfectly matches the internal consistency analysis of other validation studies. External validity was assessed by comparing the APAIS with Spielberger's State-Trait Anxiety Inventory (STAI). Moreover, found to be correlated with STAI-Y1.

Another study was conducted in 2002 to validate the Portuguese Version of (APAIS) [11]. After translation into Portuguese and administration to patients, the mean scores were: APAIS anxiety 12.82 ± 4.68, APAIS desire for information 7.33 ± 2.29, STAI-Y1 42.10 ± 10.59. Of the 109 patients, 61% were classified as anxious using STAI-Y1 (score ≥ 40), and 34% used Hospital Anxiety and Depression Scale (HADS): HADS-A and HADS-D. APAIS correlated better with the STAI-Y1 and HADS-A scores (Spearman's rho 0.580 and 0.539) than with HADS-D (Spearman's rho 0.455).

The APAIS scale is still not validated in Arabic countries and Saudi Arabia. Therefore, our research aims to translate and validate the original APAIS in Arabic and then compare it with the original version. This study aims to summarize and evaluate the validity of APAIS in Arabic, measure the prevalence of preoperative anxiety, and identify the factors associated with preoperative anxiety.

## Materials and methods

Aim & study design

This study aims to translate and evaluate the validity of APAIS in Arabic. A cross-sectional study was conducted for adaptation and Validation of the APAIS scale.

Setting & sample Size

The target population was Saudi adult patients undergoing elective surgery at the King Fahad hospital in the Qassim region of Saudi Arabia. King Fahad Hospital is dedicated to providing specialized value-based healthcare services empowered by innovation, training, and research. The minimum randomized sample size (n) is 100 patients scheduled for surgery at any department of King Fahad hospital included in this study. At the same time, patients who refuse to participate underage, patients who are not mentally stable, and critical cases are excluded.

Data collection

Data was collected in the 09/05/2022 - 01/06/2022 period. This study is both qualitative and quantitative-based. The data was collected from the open and close ending questionnaires. The close-ended questions were for a quantitative study, and the open-ended questions were for qualitative research.

Description of the measuring tool and variables

APAIS is a reliable tool to assess preoperative anxiety and determine its validity; an organized questionnaire is prepared. The questionnaire consisted of several questions based on demographic data on past surgery experience. The first part of the questionnaire consisted of personal information such as gender, age, children, and marital status. The second part of the questionnaire consisted of past medical history, including chronic conditions, previous operations, medications, type of elective surgery the patient was undergoing, and the type of anesthesia used. The last part of the questionnaire was based on the patient's experience, and their level of willingness whether they agree or not to the several questions, such as "the procedure is on my mind continually".

Ethical issues

The administration approved the study of our hospital at our institution. The research team obtained approvals from the Qassim Regional Research Ethical Committee and adhered to ethics requirements before surveying registration number 607-43-4153. The research team requested permission from King Fahad Hospital and followed all hospital's regulations while conducting this study.

## Results

Descriptive analysis was performed of the sociodemographic and underlying health conditions. Statistical Package for the Social Sciences (SPSS) performs bivariate and chi-square tests to measure central robustness and the relationship between dependable and undependable variables. P-value was determined to correlate the variables. Cronbach's Alpha coefficient > 0.7 was used to confirm the internal APAIS validity. The percentage of missing values was also used to explore the acceptability of the Arabic version among the patients.

Table 1 shows the descriptive statistics of chronic diseases and drugs. Results indicate that most of the patients (75%) are not suffering from chronic diseases such as Diabetes Mellitus, Kidney stones, Lung disorders, liver damage, etc. Only 25% of patients have chronic diseases such as Diabetes Mellitus, Asthma, HTN, Thyroid, etc., with 6%, 6%, 6%, and 7%, respectively. On the other side, drug statistics indicate that most patients (88%) are not using anxiety-related drugs, while 12% of patients are taking anxiety drugs. The descriptive statistics are done for 100 valid participants (100%).

**Table 1:** Descriptive statistics of chronic diseases and drugs

	N	Minimum	Maximum	Mean	Std. Deviation
Chronic	100	1.00	5.00	4.3900	1.20517
Drugs	100	1.00	3.00	2.0800	0.33874
Valid N (listwise)	100				

Table 2 shows the descriptive statistics of age and gender for the included (100%) patients. Results show that majority of the participants are females while less percentage of males. The period varies for all patients, and the range of age is between (13-69). The minimum age of the patient is 13, while the maximum is 69 years old. 

**Table 2:** Descriptive statistics of age and gender

	N	Minimum	Maximum	Mean	Std. Deviation
Gender	100	1.00	2.00	1.3200	0.46883
Age	100	13.00	69.00	33.8300	12.87865
Valid N (listwise)	100				

Table 3 gives the underlying health conditions of patients with a history of chronic diseases. Results show that most of the patients (75%) are not suffering from chronic diseases such as Diabetes Mellitus, Kidney stones, Lung disorders, liver damage, etc. Only 25% of patients have chronic diseases such as Diabetes Mellitus, Asthma, HTN, Thyroid, etc., with 6%, 6%, 6%, and 7%, respectively. This analysis is done for 100 participants (100%).

**Table 3:** Distribution of Chronic diseases DM- Diabetes mellitus; HTN- hypertension

		Frequency	Percent	Valid Percent	Cumulative Percent
Valid	DM	6	6.0	6.0	6.0
	HTN	6	6.0	6.0	12.0
	Asthma	6	6.0	6.0	18.0
	Thyroid disease	7	7.0	7.0	25.0
	No chronic disease	75	75.0	75.0	49.0
	Total	100	100.0	100.0	100.0

Table 4 shows the underlying surgery conditions of the patients. This surgery information will help to measure their anxiety level before surgery. Results indicate that only 56 patients (56%) had experienced the surgery in the past, while 44% of patients were new here and did not experience any surgery. This data was analyzed based on 100 patients (100%).

**Table 4:** Percentage of surgery

		Frequency	Percent	Valid Percent	Cumulative Percent
Valid	Yes	56	56.0	56.0	56.0
	No	44.0	44.0	44.0	44.0
	Total	100.0	100.0	100.0	100.0

Table 5 indicates the drug used to measure the effects of anxiety on the health status and its correlation with anxiety-related drugs. However, results show that only two percent of participants take anxiety-relieving drugs, while the majority (88%) do not use any anxiety-related drug. At the same time, 10% of patients are using other than anxiety drugs may be to treat chronic diseases. (Table 5).

**Table 5:** Medications usage

		Frequency	Percent	Valid Percent	Cumulative Percent
Valid	Anxiety drugs	2	2.0	2.0	2.0
	No drugs	88	88.0	88.0	90.0
	Other	10.0	10.0	10.0	100.0
	Total	100	100.0	100.0	

The bivariate correlation between chronic health conditions and drugs shows 0.028 significance which is <0.05, indicating a positive correlation between these two variables as most people are not experiencing any chronic disease, so they are not taking any anxiety-related drugs. Therefore, this result is the end output of this research (Table 6).

**Table 6:** Correlations of two variables *. Correlation is significant at the 0.05 level (2-tailed).

		Chronic	Drugs
Chronic	Pearson Correlation	1	0.220^*^
	Sig. (2-tailed)		0.028
	N	100	100
Drugs	Pearson Correlation	0.220^*^	1
	Sig. (2-tailed)	0.028	
	N	100	100

Table 7 shows the one-way ANOVA results to determine the patients' agreed level and whether they agreed with these statements. If yes, how much? 95% confidence interval shows that lower and upper values vary, so some patients agreed with few statements. While the rest of the patients agreed with other statements. 95% CI "I am worried about the anesthesia" shows that most of the people disagree with it, and few strongly disagree with it, which means the majority of the respondents agreed with the statement to an extent.

**Table 7:** One-way ANOVA

	Test Value = 1					
	t	df	Sig. (2-tailed)	Mean Difference	95% Confidence Interval of the Difference	
					Lower	Upper
I am worried about the anesthetic.	13.519	99	0.000	1.77000	1.5102	2.0298
The anesthetic is on my mind continually.	12.414	99	0.000	1.58000	1.3275	1.8325
I would like to know as much as possible about the anesthetic.	12.892	99	0.000	1.67000	1.4130	1.9270
I am worried about the procedure.	14.982	99	0.000	2.02000	1.7525	2.2875
The procedure is on my mind continually.	13.154	99	0.000	1.74000	1.4775	2.0025
I would like to know as much as possible about the procedure.	17.709	99	0.000	2.37000	2.1045	2.6355
Afraid	12.466	99	0.000	1.01000	0.8492	1.1708
Fear	18.024	99	0.000	2.60000	2.3138	2.8862

## Discussion

This paper is the first to explore and validate the Arabic Version of the Amsterdam Preoperative Anxiety and Information Scale (APAIS) in Saudi Arabia. Previously, its validity was determined in Spanish, German, Portuguese, and other languages. This APAIS analysis was done by 26- an item questionnaire based on three major components: demographic data, anesthesia-related concerns, and surgery-related anxiety. Results show that 96% of the respondents were from Saudi Arabia, while 4% were from other nations. However, most respondents were males at 68%, and 32% were females. The majority of respondents were between the age of 20 -50; the youngest respondent was 15 years old, while the oldest was 69 years old. Most respondents had a history of previous surgeries. The anxiety score regarding anesthesia was higher in females than males, which shows that females are more anxious than males, as shown by several studies [12,13].

The study's main objective was to check the Validation of the Arabic Version of APAIS. The methods applied during the study were according to the literature guidelines [14,15]. Strong psychometric properties were revealed during the validation process of the Arabic version concerning the reliability and validity of data. The scales used during the process showed high reliability for both anxiety sub-scale with Cronbach's alpha 0.851 and 0.827, respectively. The validity of the Arabic APAIS version was correlated with STAI-S (State-Trait Anxiety Inventory scores). For the assessment of the state of anxiety, it is considered the gold standard. Also, the original version of APAIS showed good efficiency during the configuration [16,17]. The results obtained from Arabic APAIS were similar to many studies. This Arabic APAIS differs from the Chinese and Spanish versions with a two-factor structure, whereas Chinese and Spanish have one and three factors analyzed by CFA [18]. The analysis of the data ensures the stability and validity of this scale.

In a study conducted in 2020 in China aimed to validate the Chinese Version of (APAIS) (9), they used three main steps: forward translations, blind back-translation, and pilot testing of the preliminary version. The correlation with STAI-S measured the criteria validity of the Chinese Version of APAIS, showing that the APAIS anxiety score significantly correlated with STAI-S (r = 0.717, P < 0.01. For the strategies of coping, preoperative anxiety has had a low correlation with confrontation (r = 0.33, P < 0.01) and resignation (r = 0.22, P < 0.05). So, the Chinese Version of APAIS is a valid and reliable instrument for assessing preoperative anxiety. Table 8, the 2-factor model is applied for factor analysis (F1, F2). This 2-factor model gives us a relationship between many variables and more minor variables to give the variance and eigenvalue. Results show the 45.55% and 19.84% variance and 3.61 and 2.00 eigenvalue, respectively.

**Table 8:** Communalities, factor loadings, eigenvalue, percent of the variance in a two-factor

Communalities		F1	F2	Eigenvalue	% of variance
A1	0.59	−0.60	0.31	3.61	45.55%
A2	0.71	−0.73	0.23		
A4	0.82	−0.81	0.21		
A5	0.63	−0.90	0.22		
A3	0.91	0.21	−0.94	2.00	19.84%
A6	0.69	0.28	−0.89		

The APAIS scale was further classified into two scales: one was the anxiety scale that has already been studied sufficiently; however, there are fewer data available related to the information scale dealing with the coping style of patients [19].

In this study of APAIS, the questionnaire was lengthy and time-consuming, which gave us a low response rate but good acceptability from Arabic patients. One hundred participants were asked to provide their opinions according to the scale (dichotomous) clear or unclear. A satisfactory response was obtained from all the patients with no misunderstanding. This might be because of three possible reasons. Firstly the APAIS response format was simple, colloquial, and easy to understand. Secondly, APAIS condition-specific scale relating to anesthesia and surgery was pertinent and accessible in the preoperative stage. Although no significant anxiety is seen in any patient, it seems like people have fear in their minds before undergoing anesthesia. Table 9 gives patients' worrying, fear, and nervousness before the surgery by assigning the no of anxiety levels from 1-6 based scale. Results show that people are not worried about anesthesia (1 level). Anesthesia is on the mind of patients continually to a large extent, and people are more curious to know about anesthesia. People are enough worried about this procedure because it is on their minds continually, so they are interested to know about it as much as possible.

**Table 9:** Inter-item correlation matrix

Item	1	2	3	4	5	6
1. I am worried about the anesthetic	1. 000					
2. The anesthetic is on my mind continually	0.617	1.000				
3. I would like to know as much as possible about the anesthetic	0.386	0.326	1. 000			
4. I am worried about the procedure	0.593	0.789	0.378	1.000		
5. The procedure is on my mind continually	0.497	0.389	0.426	0728	1.000	
6. I would like to know as much as possible about the procedure	0.196	0.278	0.593	0.627	0.587	1.000

According to our study results, 26% of respondents agree that they felt worried. However, we cannot find any relationship between anxiety and anesthetic in Arabic APAIS [16]. Demographic variables are not studied as in other validation studies (Spanish and German) [19]. It is important to note that perceived threat is independent of the expected risk factors as no relation is found between anxiety level and anesthesia. We tested the validity of the Arabic APAIS against the Gilliam Asperger's Disorder Scale (GADS) for this study [20]. During the study, we only faced one limitation: the questionnaire had to be completed before entering the OR (operating room). It could be possible that the level of anxiety before the surgery could variate depending on the moment of evaluation. On the other hand, we were waiting time before the surgery is an essential factor influencing the anxiety level.

## Conclusions

The Arabic version of the APAIS is a reliable and acceptable tool for measuring patients' preoperative anxiety for Arabic speaking patients. The results demonstrate the usefulness, brevity, clinical relevance, and high levels of patient acceptability of the Arabic Version of the APِِِِAIS, confirming its validity and reliability. It is also provided in a manner that makes analysis easy and quick. Due to its characteristics, this scale may be used as a standard assessment tool to quantify preoperative anxiety, particularly if verified versions are required. Much like other languages such as Japanese, Chinese, Spanish, Malaysian and Indian, etc., the APAIS could be considered validated for the Arabic version. The translation of the (English to Arabic) questionnaire made it easy for the patients to express their feelings of worry and fear efficiently and effectively. The Arabic translation made it possible to assess its validity. The translation results were clear and transparent to proceed with the research. Ultimately, this research can open the doors for future research to manage preoperative anxiety in patients.

## Appendices

**Table 10:** 6 items of the APAIS scale translated into the Arabic language

Original items	Arabic version
I am worried about the anesthetic.	.أنا قلق بشأن التخدير
The anesthetic is on my mind continually.	.التخدير في ذهني باستمرار
I would like to know as much as possible about the anesthetic.	.أود أن أعرف أكبر قدر ممكن عن التخدير
I am worried about the procedure.	.أنا قلق بشأن الإجراء
The procedure is on my mind continually.	. هذا الإجراء في ذهني باستمرار
I would like to know as much as possible about the procedure.	. أود أن أعرف أكبر قدر ممكن عن الإجراء
